# NRGsuite: a PyMOL plugin to perform docking simulations in real time using FlexAID

**DOI:** 10.1093/bioinformatics/btv458

**Published:** 2015-08-06

**Authors:** Francis Gaudreault, Louis-Philippe Morency, Rafael J. Najmanovich

**Affiliations:** Department of Biochemistry, Faculty of Medicine and Health Sciences, University of Sherbrooke, Sherbrooke, Canada

## Abstract

Ligand protein docking simulations play a fundamental role in understanding molecular recognition. Herein we introduce the NRGsuite, a PyMOL plugin that permits the detection of surface cavities in proteins, their refinements, calculation of volume and use, individually or jointly, as target binding-sites for docking simulations with FlexAID. The NRGsuite offers the users control over a large number of important parameters in docking simulations including the assignment of flexible side-chains and definition of geometric constraints. Furthermore, the NRGsuite permits the visualization of the docking simulation in real time. The NRGsuite give access to powerful docking simulations that can be used in structure-guided drug design as well as an educational tool. The NRGsuite is implemented in Python and C/C++ with an easy to use package installer. The NRGsuite is available for Windows, Linux and MacOS.

**Availability and implementation**: http://bcb.med.usherbrooke.ca/flexaid.

**Contact:** rafael.najmanovich@usherbroke.ca

**Supplementary information:** Supplementary data are available at *Bioinformatics* online.

## 1 Introduction

Docking simulations can be used to understand the specificity and selectivity of ligands as well as guide in the identification and design of inhibitors. Docking simulations seek to address three interdependent but distinct problems: (i) The prediction of the structure of a ligand-protein complex (binding mode), (ii) The discrimination of binders from non-binders (virtual screening) and (iii) The prediction of binding affinities. Docking methods are not yet successful in these three tasks simultaneously (Huang *et al.*, 2010). FlexAID was primarily developed with the task of predicting binding modes. When docking on non-native-complex structure (i.e. the structure of the target was not crystallized in the presence of the ligand of interest), FlexAID has been shown (Gaudreault and Najmanovich, 2015) to outperform existing methods such as AutoDock Vina (Trott and Olson, 2010) and FlexX (Kramer *et al.*, 1999) irrespective of target flexibility as well as rDock (Ruiz-Carmona *et al.*, 2014) when side-chain conformational changes are crucial.

PyMOL (DeLano) is a powerful, open source program for molecular visualization that allows users to extend the capabilities of the program via plugins. The source code of the latest version 1.7.6 of PyMOL is available in Sourceforge. Two PyMOL plugins exist for docking simulations (Lill and Danielson, 2011; Seeliger and de Groot, 2010), both use AutoDock Vina and require additional software compilation/installation. The latter permits docking with SLIDE (Zavodszky *et al.*, 2002) but requires an AMBER license.

A number of docking web-servers exist, including SwissDock (Grosdidier *et al.*, 2011), istar (Li *et al.*, 2014), Rosetta (Combs *et al.*, 2013) and DOCK Blaster (Irwin *et al.*, 2009) primarily focused on virtual screening. In this work we present the NRGsuite, an open-source and self-contained precompiled PyMOL plugin (for PyMOL versions 1.2 and above) focused on the prediction of binding-poses with docking simulations. The NRGsuite cohesively combines tools we develop (GetCleft and FlexAID) into an intuitive and project-oriented environment making structure-guided drug design accessible to non-experts.

## 2 GetCleft

The definition of the binding-site is important in docking simulations. Whereas in the case of single chain enzymes the binding-site lies within the largest cleft in 83% of cases (Laskowski *et al.*, 1996), the volume occupied by the binding-site within this cleft is smaller (Glaser *et al.*, 2006; Kahraman *et al.*, 2007). At a practical level, easy to use tools for the detection, refinement and measurement of the volume of buried cavities and surface clefts are not readily available. In the NRGsuite we implement the SURFNET algorithm (Laskowski, 1995) to detect surface clefts and buried cavities in proteins and nucleic acids. Our implementation of the SURFNET algorithm is called GetCleft. In short, for every pair of atoms in the macromolecule, we assess the possibility of placing a sphere midpoint between them with the largest possible radius within a user-defined range that does not overlap with the van der Walls surface of any atom. Surface exposed clefts as well as buried cavities are defined by the union of overlapping spheres and are roughly sorted by volume. The user can refine the shape of any cleft or cavity interactively and accurately measure their volumes (Supplementary Fig. S1). Users can save and utilize any original or refined cleft and cavities as target binding-sites in docking simulations. Whereas an alternative PyMOL plugin for the detection of cavities is available for Linux and Windows (Oliveira *et al.*, 2014) the resulting cavities cannot be used with FlexAID and the NRGsuite.

## 3 FlexAID

The NRGsuite interface for FlexAID contains four primary panels to define the input target and ligand to be docked, configuration of the target and ligand and simulation. Two further panels (Scoring and Genetic algorithm) give access to more advanced parameters. Each panel is briefly described in what follows.

### 3.1 Input files

Both target and ligand can be defined from the list of PyMOL objects or loaded from a previously saved NRGsuite session. Ligands can also be input with the use of SMILES strings. SMILES strings can be easily found in databases such as PubChem or ChEMBL for existing molecules or produced with chemoinformatics software. FlexAID utilizes internal coordinates and defines automatically an anchor ligand atom as the center of rotation and translation. Users can however choose to define the anchor atom themselves. Targets must be loaded into PyMOL but are not restricted to experimental structures, homology models can also be used. For further details see Gaudreault and Najmanovich (2015).

### 3.2 Target configuration

In this panel users can define the binding-site search area, for example using a cleft previously defined with GetCleft. Users can select and use more than one cleft at the same time to perform a global search when the binding-site is unknown and interactively choose binding-site flexible side chains.

### 3.3 Ligand configuration

Users have full control on ligand degrees of freedom. Whereas rotational and translational degrees of freedom are set by default, in specific situations a user may wish to restrict them. Users can manually choose individual ligand bonds as flexible. In all cases the original ligand pose can be used as reference to calculate RMSD values. Lastly, users can set distance constraints that can be used to emulate covalent docking (Duchêne *et al.*, 2014).

### 3.4 Scoring configuration

Other ligands present in complex with the target are considered by default while water molecules are ignored; again, the user may change these settings as well as the van der Walls permeability (decreasing the penalty for steric clashes). Irrespective of the inclusion of structural water molecules in the simulation, FlexAID considers solvent interactions implicitly. Users can define a solvent exclusion force or use the pairwise energy parameters considering the implicit solvent as an extra atom-type. In this panel it is also possible to change the step size used for sampling translational, rotational and internal (dihedral angles) degrees of freedom of the ligand as well as the sampling of side-chain rotameric conformations.

### 3.5 Genetic algorithm parameters

FlexAID uses genetic algorithm. A number of important parameters, notably the number of chromosomes and generations can be defined in this panel. Additionally, the number of top results that are visualized during the simulation and the frequency (in numbers of generations) to refresh the visualization can be set.

### 3.6 Simulate

Once all parameters are set, users can start the simulation and observe in real time the display of the selected number of top solutions as the simulation progresses (Supplementary Fig. S2). At the end of the simulation the top 10 results obtained and their potential hydrogen bonds with the target are displayed. Simulations can be paused, stopped or aborted. Paused simulations can be restarted and the final genetic algorithm population of solutions from stopped or completed simulations can be used to as the initial population to start a new simulation continuing where the previous one has stopped. The continuation of a simulation can only be done when all parameters remain the same except for the genetic algorithm parameters that can be changed. Users can also reload previous simulation results as well as inspect the parameters used.

## 4 Conclusions

The NRGsuite PyMOL plugin is easy to install, available for MacOS, Linux and Windows. It gives access to state-of-the-art docking simulations using FlexAID as well as the analysis of buried cavities and surface clefts using GetCleft. The NRGsuite can be used for the prediction of binding poses to understand molecular recognition and in structure-guided drug design. In our experience the NRGsuite is also an invaluable educational tool. An extensive manual is available as Supplementary Information and online at http://bcb.med.usherbrooke.ca/flexaid for up-to-date versions.

## Supplementary Material

Supplementary Data
