# Supplementary material for: NRGsuite: a PyMOL plugin to perform docking simulations in real time using FlexAID
Source: Bioinformatics. 2015 Aug 6;31(23):3856–8. doi: 10.1093/bioinformatics/btv458 (PMC4653388; doi:10.1093/bioinformatics/btv458)
Supplement: Supplementary Data [file supp_31_23_3856__index.html]

NRGsuite: a PyMOL plugin to perform docking simulations in real time using FlexAID — NRGsuite: a PyMOL plugin to perform docking simulations in real time using FlexAID — Supplementary Data 

# NRGsuite: a PyMOL plugin to perform docking simulations in real time using FlexAID

## Supplementary Data

files

- Supplementary Data - tif file
- Supplementary Data - tif file
