# Supplementary figures and images for: NRGsuite: a PyMOL plugin to perform docking simulations in real time using FlexAID
Source: Bioinformatics. 2015 Aug 6;31(23):3856–8. doi: 10.1093/bioinformatics/btv458 (PMC4653388; doi:10.1093/bioinformatics/btv458)

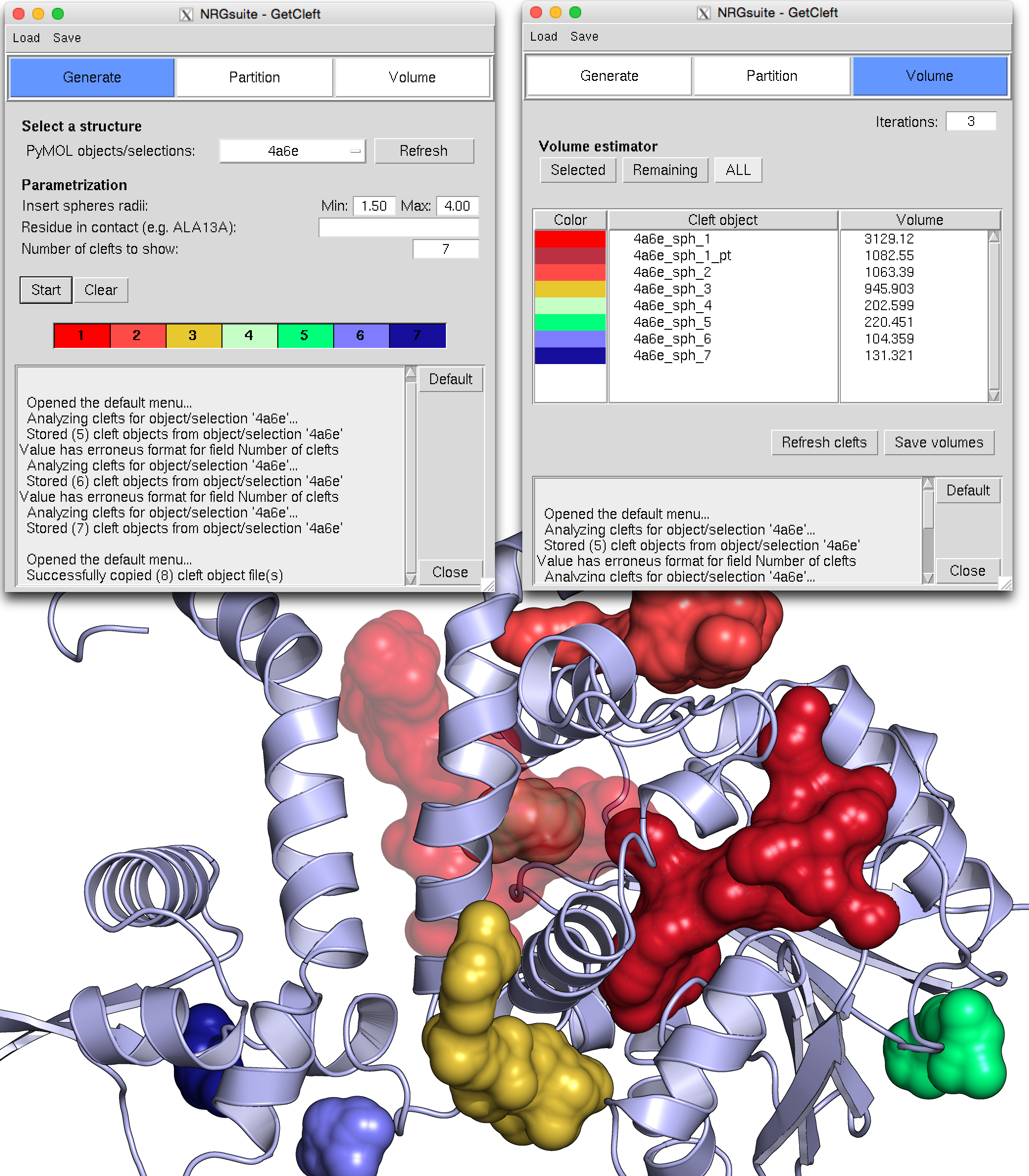

Supplement: Supplementary Data [file supp_btv458_Figure_S1.tif]

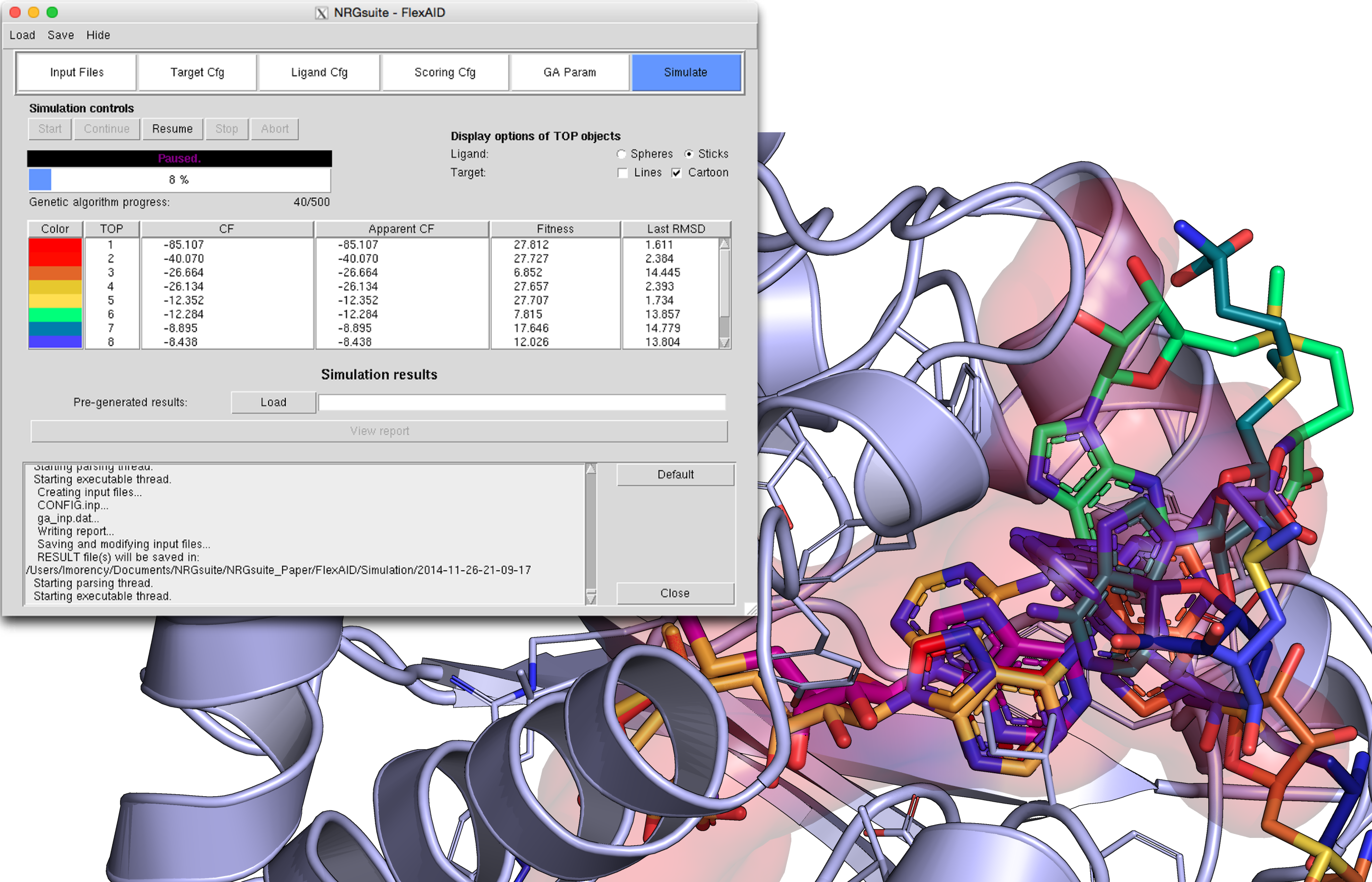

Supplement: Supplementary Data [file supp_btv458_Figure_S2.tif]
